# O016: Electronic hand hygiene monitoring for the WHO 5-moments method

**DOI:** 10.1186/2047-2994-2-S1-O16

**Published:** 2013-06-20

**Authors:** T Diller, JW Kelly, C Steed, D Blackhurst, S Boeker, P Alper

**Affiliations:** 1 Greenville Health System, Greenville SC, USA; 2 Deb Worldwide Healthcare Inc., Charlotte NC, USA

## Introduction

Two primary hand hygiene (HH) methods exist. The In/Out method teaches staff to clean their hands at entry to and exit from patient rooms. This method is easy to measure, but suffers from the potential for recontamination of staff from fomites after room entry. The World Health Organization (WHO) 5-Moments method teaches additional HH opportunities (HHOs) after entry to the patient room. It is difficult to measure and requires known direct observation. We describe the development and validation of a 5-Moments electronic monitoring system (DebMed®GMS™).

## Methods

The (DebMed®GMS™) captures soap/sanitizer dispenser activations with an implanted RF circuit board in the dispensers and transmits data via a wireless network to offsite servers. The activations represent the numerator. We previously developed an algorithm driven by patient census and nurse/patient staffing ratios to predict the number of HHOs (denominator) expected on medical/surgical, ICU or ED units (HOW2 Benchmark Study: AJIC 2011;39). The HH compliance index (HHCI) equals the activations divided by the predicted HHOs during a time period.

The HOW2 algorithm was derived from direct observation of periods of care activity, so we performed a validation study using 24 hour video-taped surveillance. We reviewed 1511 hours of video for 26 patients on a medical unit over 15 months and compared the actual HH compliance rate from the video-tape to the predicted HHCI from the (DebMed®GMS™).

## Results

Quarterly HH compliance rates by direct observation ranged from 92 to 99%. The electronic HHCI ranged from 65 to 71%, while actual HH compliance from video-surveillance ranged from 66 to 75%. Correlation of the latter two metrics was extremely high (r = 0.976, p=0.004). The number of HHOs with the In/Out method (2886) was 36% lower than with the 5-Moments method (4522).

## Conclusion

This study validates the HOW2 Benchmark Study algorithm. It also documents a 36% deficit in HHOs using the In/Out method and a ~30% Hawthorne Effect due to direct observation. There is an extremely high correlation between actual video-taped HH compliance and the electronic monitoring system’s HHCI. We believe that electronic monitoring using the 5-Moments method provides the most accurate and actionable HH compliance data.

## Disclosure of interest

T. Diller Grant/Research support from Deb Worldwide Healthcare, Inc., J. Kelly Grant/Research support from Deb Worldwide Healthcare, Inc., C. Steed Grant/Research support from Deb Worldwide Healthcare, Inc., D. Blackhurst Grant/Research support from Deb Worldwide Healthcare, Inc., S. Boeker Grant/Research support from Deb Worldwide Healthcare, Inc., P. Alper Employee of Deb Worldwide Healthcare, Inc.

